# Identification of Elements That Dictate the Specificity of Mitochondrial Hsp60 for Its Co-Chaperonin

**DOI:** 10.1371/journal.pone.0050318

**Published:** 2012-12-04

**Authors:** Avital Parnas, Shahar Nisemblat, Celeste Weiss, Galit Levy-Rimler, Amir Pri-Or, Tsaffrir Zor, Peter A. Lund, Peter Bross, Abdussalam Azem

**Affiliations:** 1 Department of Biochemistry and Molecular Biology, Tel Aviv University, Tel Aviv, Israel; 2 School of Biosciences, University of Birmingham, Birmingham, United Kingdom; 3 Research Unit for Molecular Medicine, Aarhus University Hospital, Aarhus, Denmark; Roswell Park Cancer Institute, United States of America

## Abstract

Type I chaperonins (cpn60/Hsp60) are essential proteins that mediate the folding of proteins in bacteria, chloroplast and mitochondria. Despite the high sequence homology among chaperonins, the mitochondrial chaperonin system has developed unique properties that distinguish it from the widely-studied bacterial system (GroEL and GroES). The most relevant difference to this study is that mitochondrial chaperonins are able to refold denatured proteins only with the assistance of the mitochondrial co-chaperonin. This is in contrast to the bacterial chaperonin, which is able to function with the help of co-chaperonin from any source. The goal of our work was to determine structural elements that govern the specificity between chaperonin and co-chaperonin pairs using mitochondrial Hsp60 as model system. We used a mutagenesis approach to obtain human mitochondrial Hsp60 mutants that are able to function with the bacterial co-chaperonin, GroES. We isolated two mutants, a single mutant (E321K) and a double mutant (R264K/E358K) that, together with GroES, were able to rescue an *E. coli* strain, in which the endogenous chaperonin system was silenced. Although the mutations are located in the apical domain of the chaperonin, where the interaction with co-chaperonin takes place, none of the residues are located in positions that are directly responsible for co-chaperonin binding. Moreover, while both mutants were able to function with GroES, they showed distinct functional and structural properties. Our results indicate that the phenotype of the E321K mutant is caused mainly by a profound increase in the binding affinity to all co-chaperonins, while the phenotype of R264K/E358K is caused by a slight increase in affinity toward co-chaperonins that is accompanied by an alteration in the allosteric signal transmitted upon nucleotide binding. The latter changes lead to a great increase in affinity for GroES, with only a minor increase in affinity toward the mammalian mitochondrial co-chaperonin.

## Introduction

Mitochondrial Hsp60 belongs to the family of type I chaperonins, which play a key role in mediating the correct folding of newly translated, translocated, as well as stress-denatured proteins, in mitochondria, chloroplasts and eubacteria [1]. Early studies in yeast identified the mitochondrial Hsp60 protein as essential for the folding and assembly of proteins imported into mitochondria [2], [3] as well as preventing the denaturation of mitochondrial proteins during heat-stress [4]. Due to the vital cellular functions of this protein, it is essential for viability in yeast [5] and its inactivation results in embryonic lethality in mice [6]. Moreover, mutations in this protein were discovered to be the root cause of a number of severe genetic diseases in humans [7]–[9]. In addition to the primary essential mitochondrial protein-folding activity, results of diverse studies have implicated the mammalian mitochondrial chaperonins (mHsp60 and mHsp10) in a wide range of extra-mitochondrial activities. A number of reports have suggested that mHsp60 can stimulate human leukocytes and vascular endothelial cells to produce pro-inflammatory cytokines, while mHsp10 was shown to stimulate the production of anti-inflammatory cytokines and suppress the production of pro-inflammatory cytokines [10]–[12]. Recent studies shed light on the mechanism by which mitochondrial Hsp60 is secreted from cells, enabling it to exert such extracellular functions [13]–[15]. Furthermore, mHsp60 was reported to have pro-apoptotic and anti-apoptotic roles, depending on its cellular localization [16], [17]. Finally, mHsp60 and mHsp10 were found to change their expression pattern in tumor cells [18]–[20]. Unraveling the molecular basis for these disparate functions of mHsp60 will benefit from a deeper understanding of its structural and functional properties.

**Figure 1:**
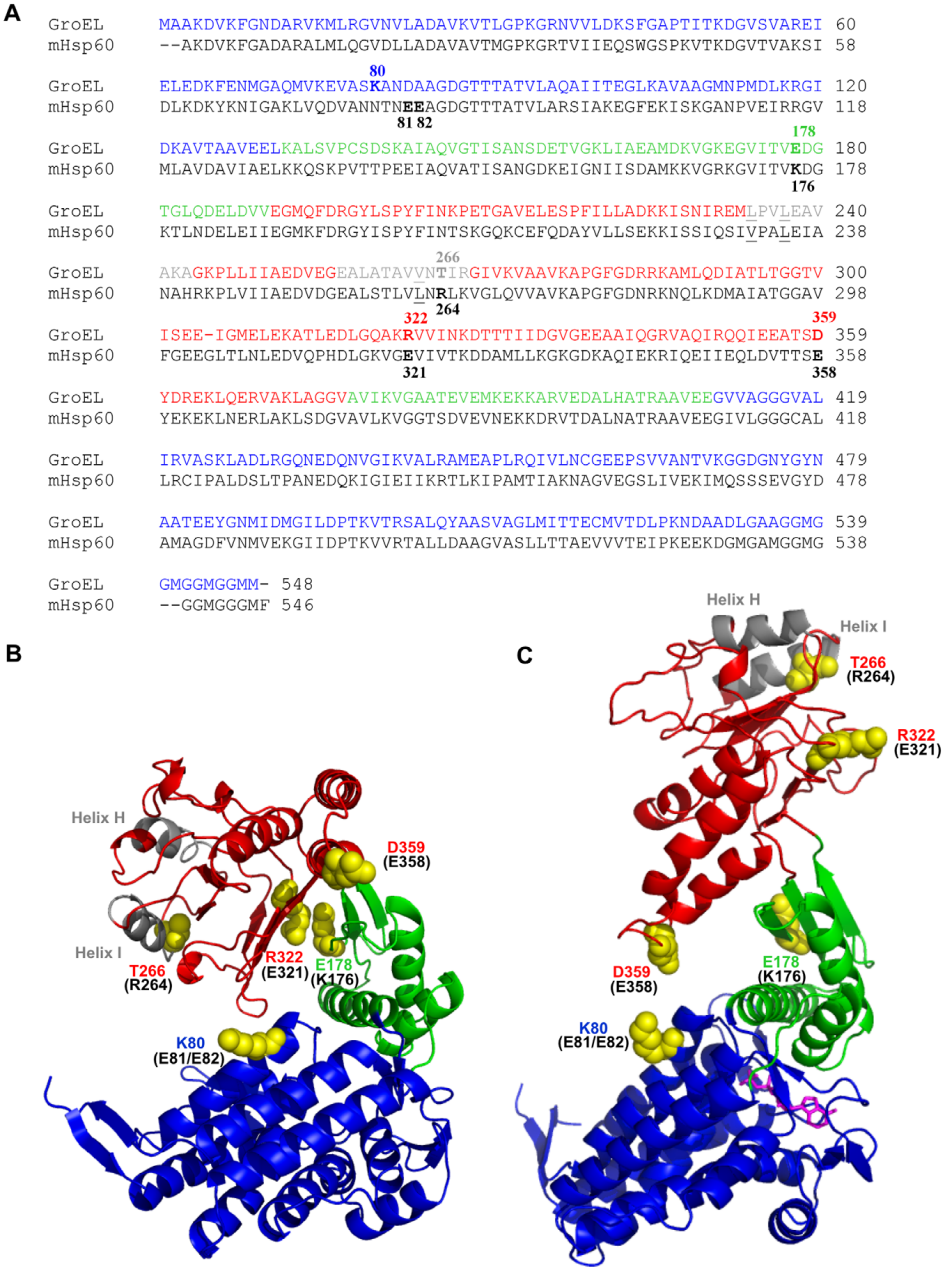
Positions of mutations in the primary and tertiary structures of GroEL. (A) Alignment of the amino acid sequences of GroEL and the mature mHsp60 protein. Protein sequence alignments were carried out by ClustalW. Amino acids discussed in this study are marked in boldface type. The amino acids known to be in direct contact with GroES are underlined. The color code corresponding to domain boundaries is described below. (B–C) 3D-structure models of GroEL subunit in the down (B) and up (C) conformations (Protein Data Bank entry 1AON) [43]. The amino acids discussed in this study are labeled and presented as space-filling models. The corresponding amino acids in mHsp60 are indicated in brackets. The ADP molecule is colored in purple. The three domains as defined by GroEL are color-coded on the GroEL sequence and structure: equatorial (blue), intermediate (green) and apical (red). Helices H and I are colored in gray. The figure was produced using PyMOL software.

Most of the mechanistic data available to date for type I chaperonin proteins comes from extensive studies carried out over the past two decades on the bacterial chaperonins (reviewed in [21]–[23]). A general picture has emerged in which the protein folding function is executed by the concerted action of two constituent oligomeric proteins, the chaperonin (known in bacteria as GroEL and in mammals as mHsp60) and the co-chaperonin (known in bacteria as GroES and in mammals mHsp10). The chaperonin oligomer is composed of fourteen identical subunits that are arranged in a barrel-like structure that is made up of two stacked heptameric rings, each enclosing a large central cavity [24], [25]. Each subunit is composed of an equatorial, an intermediate and an apical domain, the latter of which binds substrate protein and co-chaperonin [26] (Fig. 1B, C). The co-chaperonin oligomer, a heptameric molecule composed of 10 kDa subunits [27], [28], binds to the apical domain of the chaperonin in the presence of ATP via a short, unstructured, yet highly conserved region, known as the mobile loop [29], [30]. Extensive studies carried out with the bacterial system have led to a generally accepted model for chaperonin function [23], [31], [32]. In the GroEL “down” conformation (also referred to as the closed form), a denatured protein adheres to hydrophobic residues that lie on the inner surface of one of the GroEL rings (the cis ring). Subsequent binding of ATP to the cis ring induces a conformational change, which enables the binding of GroES. The latter causes a further twist and extension of the chaperonin structure resulting in an enlarged cavity which exposes hydrophilic residues (also known as the “up” or “open” conformation of GroEL). These structural changes facilitate release of the substrate protein into the enclosed cavity, where it can fold in a protected environment [22], [23], [33]. ATP hydrolysis in the cis ring, and subsequent binding of ATP and GroES to the opposing (trans) ring, facilitate the release of GroES, ADP and folded protein.

Although this general concept for chaperonin function seems to be similar for all type I chaperonins, certain critical differences at the mechanistic level are known to distinguish the mitochondrial chaperonin from the chloroplast and bacterial homologs [34]–[36]. Despite the fact that mHsp60 is capable of complementing a bacterial GroEL depletion strain, when co-expressed with mHsp10 [37], it is incapable of functionally interacting with [38], or even binding to [39], GroES, the bacterial co-chaperonin. It was additionally shown that mHsp60 is not functional with co-chaperonins of plant or phage origin [34], [37]. In contrast, the E. coli chaperonin can facilitate folding with co-chaperonins from any source [40], [41]. Interestingly, despite the fact that chloroplast chaperonin β subunits are nearly identical in sequence, homologous oligomers composed of all one subtype exhibit differential activity with various co-chaperonins [42]. It was suggested that the specificity for mHsp10 stems from a generally weak affinity of mHsp60 for co-chaperonins relative to GroEL, with a correspondingly high binding affinity of mHsp10 to chaperonins compared to other co-chaperonin homologs [34]. The elements of mitochondrial chaperonins that define their specificity for co-chaperonin binding seem to lie in the apical domain, as a chimeric protein, in which the whole apical domain of GroEL was replaced with that of mHsp60, showed the same preference for mHsp10 co-chaperonin as the wild-type mHsp60 [35]. On the other side of the equation, it was previously shown that three substitutions in the GroES mobile loop, which make it similar to the loop of mHsp10, are sufficient to allow the bacterial co-chaperonin to bind and functionally interact with the mammalian mitochondrial chaperonin [34]. Although the above studies suggest that the specificity of mitochondrial chaperonin for its co-chaperonin is most likely governed by residues in mHsp60 that come into direct contact with the mobile loop, it is possible that other factors may play a role in determining the strict specificity of mHsp60 for its co-chaperonin.

Based on the ability of the mHsp60-mHsp10 pair to complement a depletion of the bacterial chaperonins in *E. coli*, we developed a screen which uses selective pressure to isolate mHsp60 mutants that are able to function when assisted by the bacterial co-chaperonin, GroES. In this study, we report the isolation and characterization of two mHsp60 mutants that are able to function with GroES. Our results show that the interaction between chaperonin and co-chaperonin is affected not only by amino acids involved in direct contact of the two proteins, but also by structural elements that are far from the binding site. Together, these factors allow for a fine-tuning of chaperonin affinity for its cofactor and determine the specificity of mHsp60 to mHsp10. The mechanistic implications of this analysis on our understanding of the wild-type mammalian mitochondrial chaperonin system are discussed.

## Results

### Isolation of mHsp60 Mutants Able to Functionally Interact with GroES

Over a decade ago, the crystal structure of a GroEL-GroES complex was solved at 3 Å resolution, allowing for visualization of the contact sites between the two oligomers. Close inspection of the structure revealed that three amino acids in the GroES mobile loop (IVL) make contact with three residues L234, L237 and V264, located in the H and I helixes of the GroEL apical domain [43]. These residues in GroEL correspond to V232, L235 and L262 in mHsp60, respectively (underlined amino acids in Fig. 1A). In order to analyze the significance of this interaction for co-chaperonin specificity of the human mHsp60, we mutated the two corresponding amino acids that differed in mHsp60, making them identical to those of GroEL. However, the purified mHsp60 mutant (V232L/L262V) exhibited wild-type protein-folding behavior and was still functional only with mHsp10 (not shown). This result suggests that, although the mutated amino acids are seen to interact with GroES in the crystal structure of the bacterial complex, other important elements must be involved in directing the specificity of the chaperonin-co-chaperonin interaction in the mitochondrial system.

**Figure 2:**
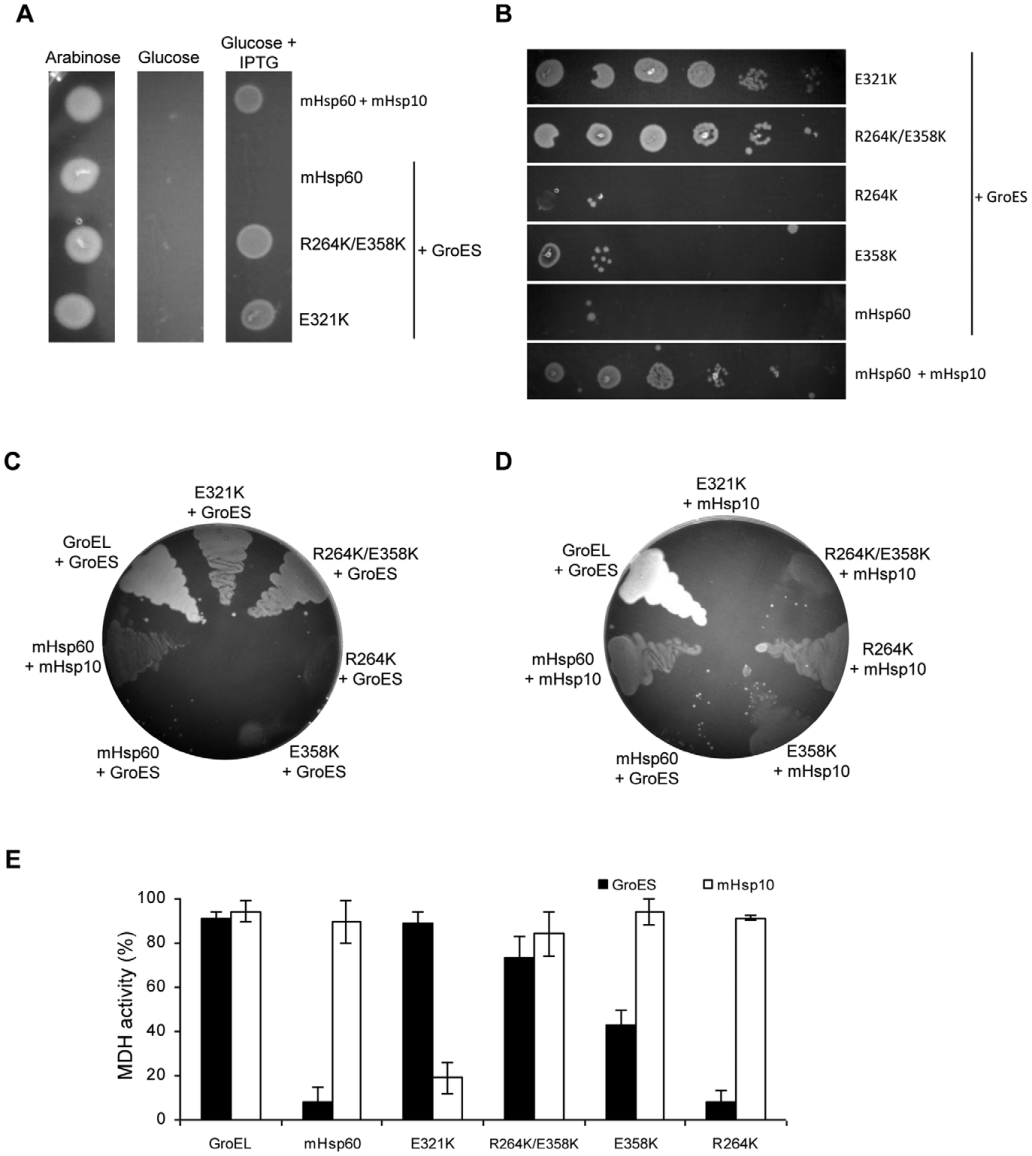
Identifying mHsp60 mutants that are functional with GroES. (A) Examination of the *in vivo* system at the indicated growth conditions. (B) Ten-fold-serial dilutions of *E. coli* strain MGM100 harboring plasmid pOFX with the indicated mHsp60 variant and GroES, grown on agar plates in the presence of glucose and IPTG. (C–D) Examination of the ability of mHsp60 mutants to facilitate the growth of MGM100 on agar plates containing glucose and IPTG in combination with GroES (C) or mHsp10 (D). GroEL-GroES and mHsp60-mHsp10 combinations serve as positive controls; the mHsp60-GroES combination serves as negative control. (E) Refolding of 0.33 µM HCl-denatured MDH by 10 µM of the indicated chaperonin and 40 µM of mHsp10 (white columns) or GroES (black columns). MDH activity was measured at 340 nm following 120 min incubation at 30°C in the presence of 1 mM ATP. The activity following refolding is presented relative to that of native MDH (100%).

In order to elucidate the molecular basis for the exclusive interaction of mHsp60 with its co-chaperonin, mHsp10, we adopted an unbiased directed evolution approach to identifying the amino acids responsible for this specificity. We developed an *in-vivo* screen for isolating mHsp60 mutants that are able to functionally interact with GroES, the bacterial co-chaperonin. Selective pressure was exerted by providing GroES as the only available co-chaperonin, thereby allowing only compatible mutants to survive (a schematic depiction is presented in Fig. S1). While we expected to find significant changes in the sequence of mHsp60 that had become functional with GroES, the mutations in the three colonies that we isolated were minor: One colony harbored only a single mutation, E321K, and the other two colonies contained identical changes which consisted of a double mutation, R264K and E358K (Fig. 2A). Notably, while all the mutated amino acids correspond to GroEL amino acids that are located in the apical domain, none of them would be expected to directly interact with the mobile loop of the co-chaperonin, based on the crystal structure of the GroEL-GroES complex [43]. T266 of GroEL, corresponding to R264 of mHsp60, is the position closest to the bound co-chaperonin, however, it emerges from the side of helix I opposite to that which is seen to make contact with the mobile loop (Fig. 1C). Interestingly, while bacteria expressing the mHsp60 mutants together with GroES exhibited significantly better growth than wild- type mHsp60 with mHsp10 (Fig. 2B & C), the amino acid changes in both mutants were detrimental to bacterial growth when expressed with mHsp10: in the case of the double mutant R264K/E358K, bacterial growth was significantly slower than wild-type, and in the case of the single mutant E321K, the bacteria were not viable (Fig. 2D). Thus, the isolated mHsp60 mutants exhibited altered specificity, with a preference for GroES over mHsp10 *in vivo*.

Following identification of the two mutants from the screen, our next goal was to elucidate the molecular mechanism by which these mutations altered the specificity of mHsp60 for co-chaperonin. To this end, the mutated proteins were expressed in bacteria, purified as oligomers and were subjected to structural and functional *in vitro* analysis.

### E321K Forms a Highly Stable Complex with mHsp10

As a first step in characterizing the mutant proteins, we examined the ability of E321K to refold HCl-denatured malate dehydrogenase (MDH) *in vitro* together with mHsp10 and GroES. As shown in Figure 2E, GroEL and wild-type mHsp60 behaved as would be expected based on previous studies: the former was active with both mHsp10 and GroES, while the latter was active only with the mitochondrial co-chaperonin, mHsp10. As opposed to the wild-type protein and in agreement with the *in vivo* results, E321K was able to facilitate the refolding of MDH only with the assistance of bacterial co-chaperonin, GroES, and not with its co-chaperonin, mHsp10. Thus, the *in vivo* and the *in vitro* results indicate that the single point mutation, E321K, switched the co-chaperonin specificity of mHsp60, from being functional only with mHsp10 to being functional only with GroES. One possible explanation for this observation could be that the E321K mutation specifically impairs the ability of mHsp60 to bind mHsp10. A second possible explanation could be that the E321K mutation causes a general increase in the binding affinity of mHsp60 to all co-chaperonins, thereby enabling the mutant to bind GroES with moderate affinity, but leading to a very tight and non-functional binding to mHsp10, which was suggested to have a relatively high affinity for chaperonins compared to other co-chaperonins [34]. Consequently, a very stable, non-functional complex between E321K and mHsp10 is formed, as opposed to the dynamic and functional complex that is formed between the wild-type mHsp60 and mHsp10.

**Figure 3:**
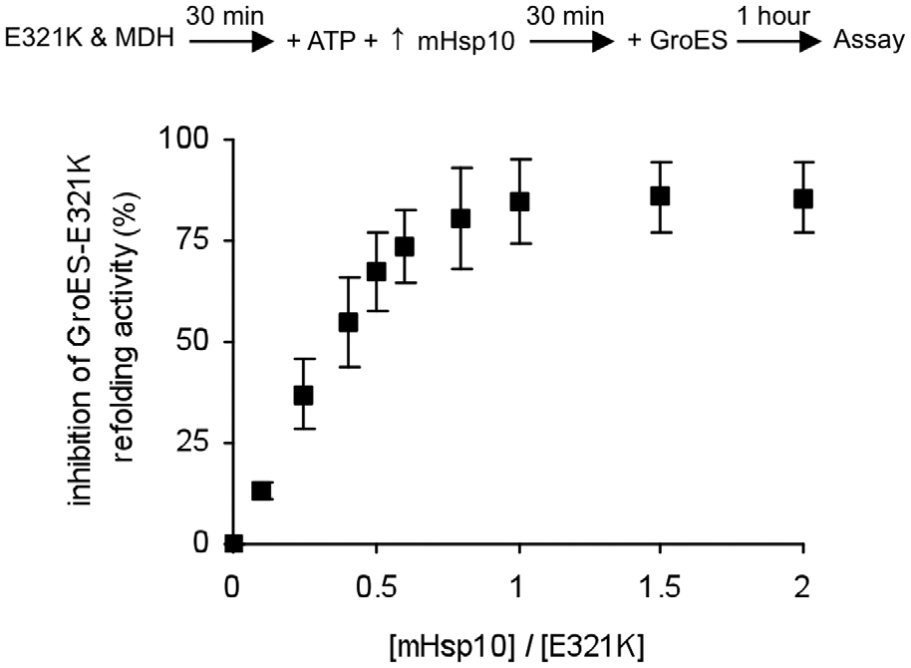
Inhibition of GroES-E321K refolding activity by mHsp10. A binary complex of E321K and HCl-denaturated MDH was pre-incubated for 30 min in the presence of increasing concentrations (from 0 to 20 µM) of mHsp10 and 2 mM ATP before adding 20 µM GroES. MDH activity was measured 1 hour following the addition of GroES. % inhibition = 100*[(A_o_–A_i_)/A_o_]. A_o_ represents the activity level in the absence of mHsp10, and A_i_ represents the activity level at each mHsp10 concentration.

**Figure 4:**
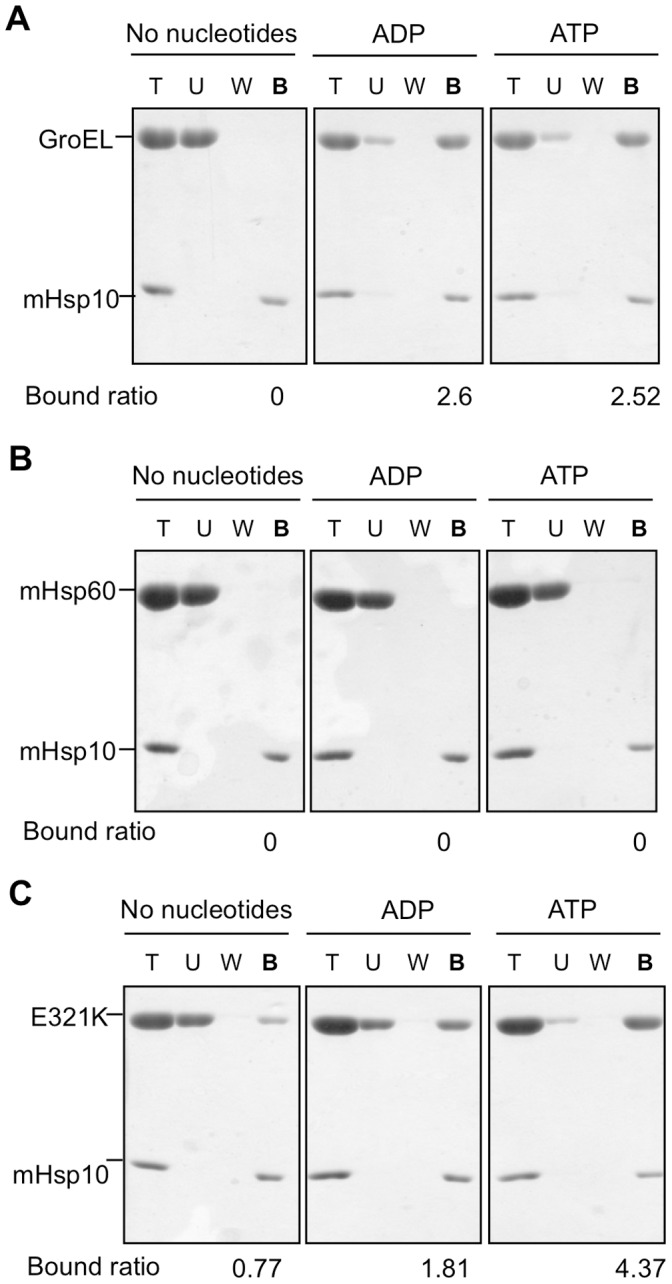
A stable complex is formed between the E321K mutant and mHsp10. Interaction between mHsp10 and different chaperonins was measured using a pulldown assay. 50 µM of His-tagged mHsp10 together with 50 µM of GroEL (A), mHsp60 (B), or E321K mutant (C) were incubated with nickel beads in the absence of nucleotides, or in the presence of 4 mM ATP or 4 mM ADP. Equivalent aliquots of 2 µl from the total sample (T), unbound fraction (U), fourth wash (W), and bound fraction (B) were analyzed by SDS-PAGE and stained with Coomassie blue. The intensities of the bands were quantified by densitometry (ImageMaster 1D Prime program). The bound ratio listed on the bottom of each gel represents the ratio between the intensities of the chaperonin and co-chaperonin bands in the bound fraction.

We employed several methods in order to experimentally probe the molecular basis for the *in vivo* behavior and *in vitro* refolding results of the mutant chaperonins. In the first, MDH refolding activity by E321K-GroES was tested following pre-incubation with the competing mHsp10. Under these conditions, refolding was profoundly inhibited by mHsp10 in a concentration-dependent manner (Fig. 3). Maximal inhibition was obtained at a mHsp10:E321K ratio of 1∶1, supporting the suggestion that a very stable complex is formed between E321K and mHsp10. We also carried out a pull-down assay using a hexa-histidine-tagged mHsp10, in order to examine whether a stable interaction could be observed between mHsp10 and either E321K, wild-type mHsp60 or GroEL. The GroEL control was detected in complex with mHsp10 only in the presence of nucleotides (Fig. 4A), consistent with previous reports [44]–[47]. For the mHsp60-mHsp10 pair, previous studies showed that mammalian mHsp10 binds to mHsp60 only in the presence of ATP, but not ADP [35]. However, in our pull-down experiment, no complex between mHsp60 and Hsp10 was observed under any conditions, even when ATP was present (Fig. 4B). The latter finding confirms that the complex of mHsp10 with mHsp60 is much more labile than the complex it forms with GroEL. In contrast, a complex between E321K and mHsp10 was observed under all condition tested, even in the absence of nucleotide (Fig. 4C). This again supports the idea that the complex formed between E321K and mHsp10 is less dynamic than the complex formed between the wild-type proteins and suggests that E321K adopts a conformation that enables it to interact with co-chaperonin, even without nucleotide binding.

**Figure 5:**
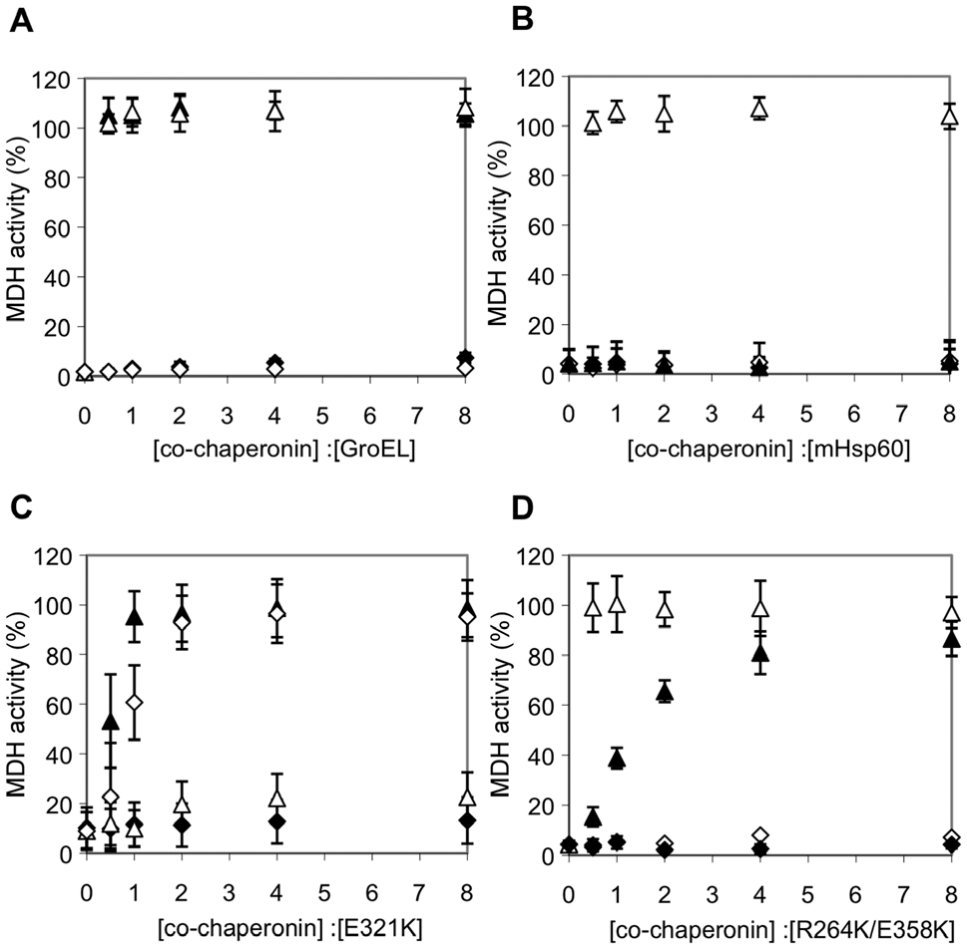
Refolding activity in the presence of increasing concentrations of wild-type and low-affinity co-chaperonins. Refolding of 0.33 µM HCl-denatured MDH by 10 µM of chaperonin GroEL (A), wild-type mHsp60 (B), E321K mHsp60 (C), R264K/E358K mHsp60 (D), in the presence of increasing concentrations of mHsp10 (white triangles), GroES (black triangles) and the low-affinity mutants: mHsp10_L33A (white diamonds) and GroES_L27A (black diamonds). MDH activity was measured at 340 nm following 120 min incubation at 30°C in the presence of 1 mM ATP. The 100% reference was determined as the activity of a sample containing the same amount of native MDH.

We hypothesized that if an increased affinity of the E321K mutant to mHsp10 is responsible for the lack of activity, the function might be rescued by replacing wild-type mHsp10 with a “low affinity” mutant. Leucine 27, located in the mobile loop of GroES, is a highly conserved residue among co-chaperonins that was shown to contact GroEL in the crystal structure [43]. Consistent with this, it was previously shown that the L27A mutant of GroES cannot interact with GroEL [39]. In this study, we created the corresponding mutation in mHsp10 (L33A). Neither GroEL (Fig. 5A) nor wild-type mHsp60 (Fig. 5B) was able to refold denatured MDH when incubated with either of the “low-affinity” co-chaperonin mutants. In contrast, the E321K mutant of mHsp60 was active with the mHsp10 mutant, L33A, but not with the GroES mutant, L27A (Fig. 5C). Thus, our MDH-refolding results clearly show that the E321K mutation of mHsp60 can compensate for the decreased affinity of the L33A mHsp10 mutant to chaperonins.

The ability of co-chaperonins to inhibit ATP hydrolysis activity of chaperonins is another measure of their interaction. It is well known that upon binding of co-chaperonin, the ATPase activity of the chaperonin is inhibited by ∼50% [31], [48]. In our system, the ATPase activity of GroEL was inhibited by both GroES and mHsp10 (by 67% and 53%, respectively) and that of mHsp60 was inhibited, as expected, only by mHsp10 (by 49%) (Table 1). The two low-affinity co-chaperonin mutants did not inhibit the ATPase activity of GroEL or of wild-type mHsp60, which suggests that no binding occurs between these pairs. In the case of the E321K mutant, the only co-chaperonin that did not inhibit the ATPase activity was the L27A GroES mutant, the same mutant which did not assist in refolding. Notably, of all combinations examined, the E321K-mHsp10 exhibited the highest inhibition of ATPase activity (84%), again suggesting that a stronger binding takes place between these two proteins (Table 1).

**Table 1:** Inhibition of chaperonin ATPase activity by various co-chaperonins.

	ATPase inhibition (Refolding activity)			
	GroEL	mHsp60	E321K	R264K/E358K
**mHsp10**	53% (+)	49% (+)	84% (−)	48% (+)
**GroES**	67% (+)	0% (−)	30% (+)	47% (+)
**mHsp10_L33A**	0% (−)	0% (−)	26% (+)	40% (−)
**GroES_L27A**	0% (−)	0% (−)	0% (−)	0% (−)

Steady-state ATPase activity was measured for each chaperonin. The T.O.N values (1/min) were 3.27±0.32, 0.83±0.14, 0.91±0.1 and 0.79±0.08 for GroEL, mHsp60, E321K and R264K/E358K, respectively. The percentage of ATPase inhibition by each co-chaperonin is indicated. The experiment was carried out using a 2∶1 molar ratio of co-chaperonin:chaperonin. Plus (+) and minus (−) indicate the ability and inability, respectively, of each chaperonin-co-chaperonin pair to mediate the refolding of HCl-denaturated MDH (as depicted in Fig. 5).

**Figure 6:**
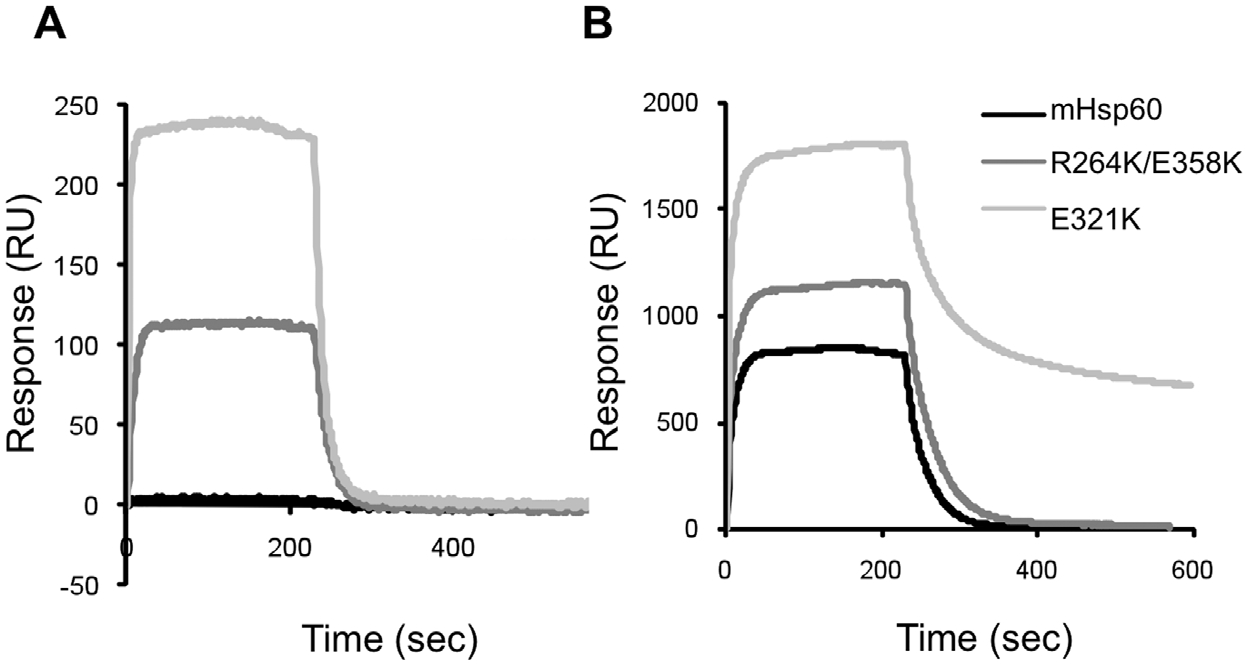
Chaperonin-co-chaperonin interactions measured by SPR. Association and dissociation patterns of 10 µM of the indicated chaperonin to immobilized (A) GroES (∼ 600 Relative Units-RU) or (B) mHsp10 (∼ 800 RU) in the presence of 2 mM ATP.

All the above-mentioned results support the hypothesis that the E321K mutant was able to function with GroES due to a general increase in binding affinity for co-chaperonins. In order to obtain direct support for this hypothesis, we used surface plasmon resonance (SPR) to evaluate the association equilibrium constant (K_A_) in a direct manner (Fig. 6 and Table 2). As expected, no binding was observed between wild-type mHsp60 and GroES, while E321K showed significant binding to GroES. In support of the increased-affinity hypothesis, a 6-fold increase in the apparent K_A_ value was measured for the association of mHsp10 with E321K, compared to its association with wild-type mHsp60. Notably, the tight binding between E321K and mHsp10 is also reflected in the distinct dissociation pattern of this protein complex (Figure 6B). The bi-phasic dissociation pattern of E321K from mHsp10 can be explained by the concurrence of two distinct processes: monomerization of the unstable mHsp60 oligomers [49] and slow dissociation of a very stable and non-functional complex formed between E321K and mHsp10.

**Table 2:** SPR analysis.

Association equilibrium constant (K_A_) ratio^1^			
	mHsp60	R264K/E358K	E321K
**mHsp10**	1±0.19	1.53±0.22	5.97±0.60
**GroES**	ND^2^	0.62±0.12	1.43±0.31

1 Ratios of association equilibrium constant represent the apparent K_A_ measured between each pair relative to the apparent K_A_ measured between mHsp60 and mHsp10 (apparent K_D_ of 7.4 µM). The apparent values of K_A_, the association constant (M^−1^), were determined using equilibrium analysis [65], [66]. Values represent average ± SEM of at least three independent experiments.

2 ND, no binding detected.

### Structural Interpretation for the E321K Phenotype

The finding that E321K creates an inactive, very stable complex with mHsp10, led us to seek the structural basis underlying this observation. It was previously suggested that residue R322 of GroEL creates a salt-bridge with E178 in the down conformation of GroEL [24] (Fig. 1B), but not in the up, co-chaperonin bound conformation [43], [50] (Fig. 1C). It was proposed that mutations abolishing the formation of this salt-bridge should strengthen the interaction with co-chaperonins by allowing the molecule to reach the up conformation more easily [51], [52]. Although residues 322 and 178 are not well conserved among chaperonins, the coupling of positively- and negatively-charged amino acids, potentially forming this salt bridge, is highly conserved from bacterial to mitochondrial chaperonins in corresponding positions (Fig. S3). Indeed, the homologous amino acids to GroEL’s R322 and E178 are E321 and K176 in mHsp60, respectively.

**Figure 7:**
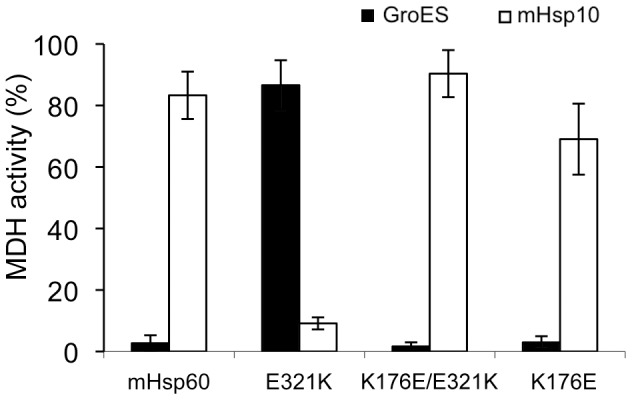
The effect of the K176E mutation on the function of mHsp60 with co-chaperonins. Refolding of 0.33 µM HCl-denatured MDH by 10 µM of the indicated chaperonin and 20 µM of mHsp10 (white columns) or GroES (black columns). MDH activity was measured at 340 nm following 60 min incubation at 30 °C in the presence of 1 mM ATP. The activity following refolding is presented relative to that of native MDH (100%).

In order to experimentally probe the hypothesis that the breakdown of this salt bridge is responsible for the “high-affinity” phenotype of the E321K mutant, we mutated the putative salt-bridge partner of E321 in mHsp60, namely K176. The K176E mutation was tested as a single mutant, on the background of the wild-type protein, and as a double mutation on the background of the E321K mutation (Fig. 7). As would be expected, the K176E/E321K double mutant, in which the salt-bridge between these amino acids is predicted to be restored, was active only with mHsp10, and not with GroES, similar to the wild-type mHsp60. However, the K176E single mutant, which would be expected to exhibit a high affinity phenotype, similar to the E321K mutant, was also functional only with mHsp10, like the wild-type protein. It is possible that in the K176E mutant, an alternative salt bridge is formed between one of these negatively charged positions with another, positively-charged amino acid, thereby restoring the wild-type phenotype.

### Properties of the Double Mutant R264K/E358K

The second clone of mHsp60 that was isolated in our screen carries the mutations R264K and E358K, and was able to complement GroEL *in vivo*, together with either GroES or mHsp10. Surprisingly, although bacterial growth was observed to be slower in the presence of mHsp10 compared to the growth in the presence of GroES (Fig. 2C & D), the MDH refolding yields *in vitro*, were actually higher with mHsp10 than with GroES (Fig. 5D). The better *in vitro* functionality of the double mutant with mHsp10 is emphasized by the fact that much higher GroES concentrations are needed to reach maximum yield of refolded MDH. While the double mutant together with mHsp10 reaches maximum activity at a low co-chaperonin: chaperonin ratio of 0.5∶1, maximal yield in the presence of GroES was achieved only at a ratio of ∼4∶1.

In order to examine the contribution of each individual mutation to the observed phenotype, we created two additional constructs of mHsp60, one carrying the R264K mutation and the second carrying the E358K mutation. The three mutants, R264K/E358K, R264K and E358K, were similarly active with mHsp10 *in-vitro* (Fig. 2E), despite the differences that are observed when they are co-expressed with mHsp10 *in-vivo* (Fig. 2D). When the single mutants E358K or R264K were co-expressed with GroES, little, if any, bacterial growth was observed (Fig. 2C). *In-vitro*, in the presence of GroES, the R264K mutant exhibited only background refolding activity, like wild-type mHsp60, while the E358K mutant exhibited approximately half of the activity displayed by the double mutant (Fig. 2E). These *in-vivo* and *in-vitro* results indicate that mutations R264K and E358K somehow act in a synergistic manner to allow the double mutant to function with GroES.

### The R264K/E358K Mutant Exhibits Differential Increase in its Affinity for Co-Chaperonins

Does the double mutant R264K/E358K operate, as in the case of the single mutant E321K, mainly by a mechanism of global increased affinity, or via a distinct mechanism? The ability to functionally interact with mHsp10 *in-vivo* and *in-vitro* (Fig. 2D & E), together with the higher concentrations of GroES needed for maximal refolding activity *in-vitro* (Fig. 5D), suggest that the double mutant has lower affinity for co-chaperonin compared to the E321K mutant. As a first step in analyzing the mechanism for activity of the double mutant with GroES, we tested its ability to function with the low-affinity mHsp10 mutant, L33A. We found that, although the mHsp10 mutant L33A was able to bind to the mHsp60 double mutant, based on its ability to inhibit the ATPase activity (Table 1), this interaction did not lead to a functional complex (Fig. 5). Thus, the L33A mutant binds the double mutant but is incapable of facilitating MDH-refolding activity. These results are consistent with previous studies showing that not all interactions between chaperonin and co-chaperonin lead to productive folding [39], [53].

Finally, binding of the double mutant R264K/E358K to co-chaperonins was evaluated using surface plasmon resonance (SPR). Consistent with the *in vivo* data (Fig. 2C–D) and the functional *in vitro* data (Figs. 2E and 5D), the double mutant demonstrated binding to both GroES and mHsp10. Quantitatively, the double mutant exhibited only a 1.5-fold increase in the apparent K_A_ value to its mitochondrial partner co-chaperonin, compared to the wild-type protein (Table 2). We hypothesize that such a small increase in the affinity between the double mutant and the co-chaperonin might not be sufficient by itself to explain the gain of function between mHsp60 and GroES.

### Effect of ADP on Protein Refolding by mHsp60 Mutants

**Figure 8:**
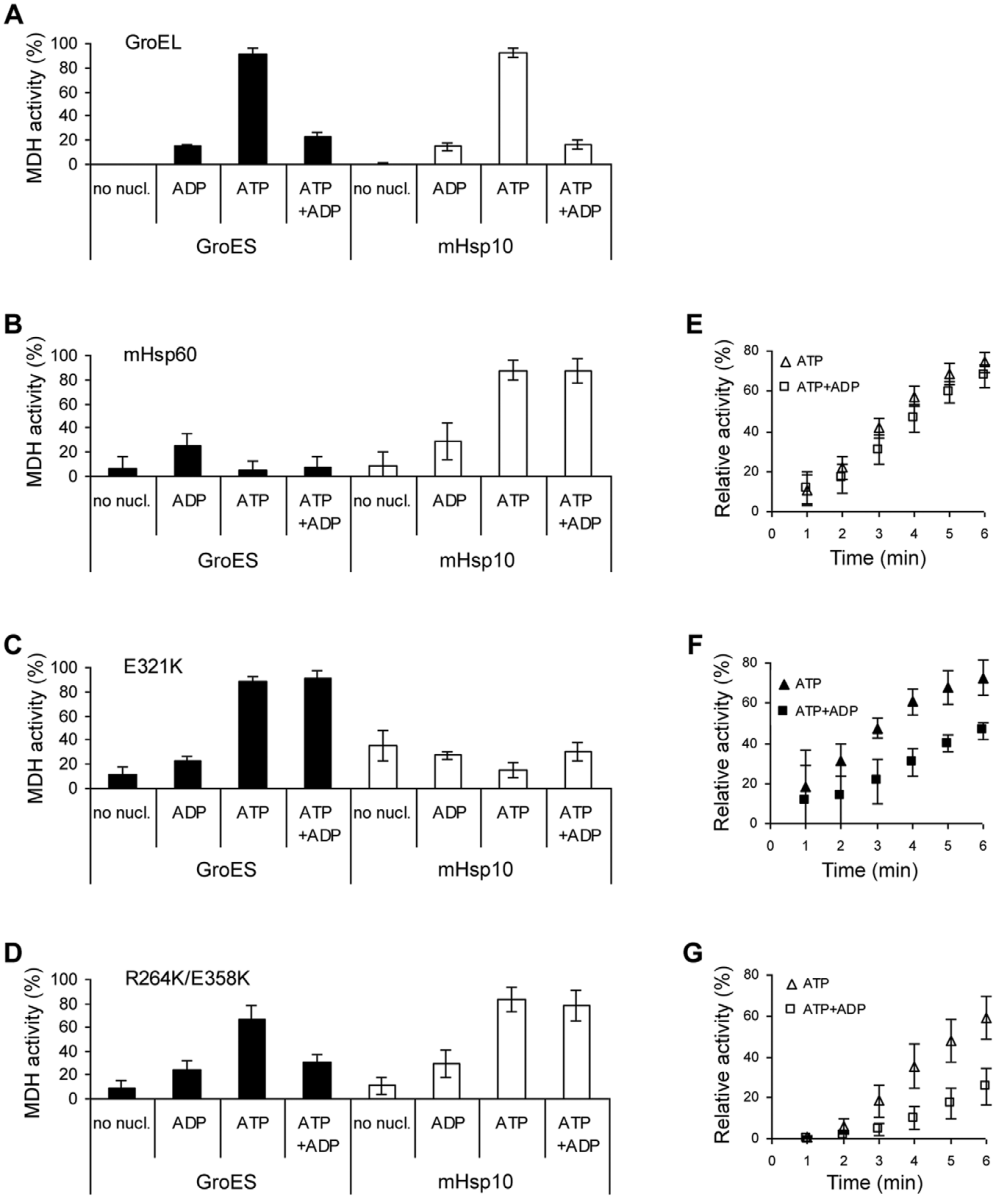
The inhibitory effect of ADP on MDH refolding activity by chaperonins. (A–D) Refolding of 0.33 µM HCl-denatured MDH by 10 µM of the indicated chaperonin and 40 µM of mHsp10 (white columns) or GroES (black columns). MDH activity was measured at 340 nm following a 60 min incubation at 30°C in the absence of nucleotides or in the presence of 10 mM ADP, 1 mM ATP or 1 mM ATP+10 mM ADP as indicated. The activity following refolding is presented relative to that of native MDH (100%). (E–G) Time-dependent refolding activity of wild-type mHsp60 (E), E321K mutant (F) and R264K/E358K mutant (G) together with mHsp10 (white symbols) or GroES (black symbols) in the presence of 1 mM ATP (triangles) or 1 mM ATP+10 mM ADP (squares). The relative activity is compared to the activity measured by each chaperonin pair after 30 min in the presence of ATP (100%).

It is well known that ADP acts as a strong inhibitor of both ATP hydrolysis and protein folding activity of GroEL, by competing with ATP for nucleotide binding sites [54], [55]. Consistent with this, when a ten-fold excess of ADP over ATP was included in the reaction mixture, the yield of MDH refolding activity of GroEL with either mHsp10 or GroES was inhibited by ∼75% (Fig. 8A). In contrast, in the case of wild-type mHsp60, no inhibition of MDH refolding activity was observed in the presence of excess ADP over ATP (Fig. 8B), even though it was previously shown that a 5-fold excess ADP over ATP inhibits the ATPase activity by ∼50% [36], indicating that ADP does bind to mHsp60.

Since the effect of ADP is related to the allosteric behavior of chaperonins [55], we sought to determine whether ADP affects protein folding activity of the two mHsp60 mutants. As shown in Fig. 8D, ADP was able to significantly inhibit the yield of refolded MDH by the double mutant, when assisted by GroES, but not when assisted by mHsp10. Notably, the yield of the refolded MDH by E321K-GroES pair was not inhibited by ADP (Fig. 8C). For those cases, in which ADP did not affect the refolding yields, we examined whether a more modest effect could be detected on initial refolding rates. Indeed, excess ADP over ATP decreased the initial refolding rates by the double mutant-mHsp10 (Fig. 8G) and E321K-GroES (Fig. 8F) pairs. However, no inhibition by ADP was detected at any level for the wild-type mHsp60-mHsp10 pair (Fig. 8E). These results suggest that the isolated mutants exhibit some alterations in the nucleotide binding properties and/or in the transmission of allosteric signals, in such a manner that ADP becomes an inhibitor of their function under our experimental conditions.

## Discussion

Chaperonin proteins, mHsp60 and mHsp10, are key players in the homeostasis of mitochondria since they mediate the folding of proteins in the matrix, an environment containing only a limited number of chaperones (in the human mitochondria there are only one Hsp60, one Hsp70 and no ClpB homologues) [56]. In addition to their chaperonin function, it is well documented that mHsp60 and mHsp10 affect processes that are not related directly to protein folding, such as apoptosis [16], [17] and inflammation [10]–[12]. One may assume that the diverse repertoire of functions affected by the mitochondrial chaperonin system will be reflected in significant structural differences when compared to the bacterial chaperonin and co-chaperonin homologs. For example, it could be that the monomeric form, not the tetradecameric form, is functional outside mitochondria or cells. In such a case the cooperation with Hsp10 will not be required. The specificity that mHsp60 has developed for the mitochondrial co-chaperonin could be explained by such structural drift in these molecules. We used a directed evolution approach to elucidate the structural basis for chaperonin-co-chaperonin specificity in the mitochondrial system and isolated two mHsp60 mutants that were able to function with GroES, a single mutant E321K and a double mutant R264K/E358K. The fact that amino acids distant to the contact site have such a dramatic effect on activity reflects the importance of dynamic transitions for function of the system. Subsequent experiments were carried in order to determine the molecular basis for the gain of GroES-dependent chaperone function in these two mHsp60 mutants.

### The E321K Mutant

Both the *in vivo* and the *in vitro* refolding results showed that the E321K mutant switched its co-chaperonin functional specificity. In contrast to the wild-type mHsp60, that could function only with its natural co-chaperonin partner, mHsp10, the E321K mutant could function only with the assistance of the bacterial co-chaperonin, GroES. Pull-down, folding, ATPase and SPR experiments all suggest that the substitution of glutamate by lysine in E321K caused a significant increase in the affinity of the chaperonin protein to all co-chaperonins. This global increase in affinity enabled the mutant mHsp60 to functionally interact with the low-affinity co-chaperonin, GroES, and with the low affinity mHsp10 mutant, L33A. However, this same increase in affinity resulted in the formation of an inactive, very stable complex between mHsp10 and E321K, which in contrast to other chaperonin-co-chaperonin complexes, did not require nucleotide for its formation (Fig. 4). The results with E321K support a previous proposition that increasing the affinity of mHsp60 for co-chaperonin will enable the protein to function with GroES [52]. Our results are also consistent with previous studies which demonstrated that GroEL mutants that acquire the ability to function with a GroES temperature sensitive mutant (G24D) are impaired in their ability to function with wild-type GroES [52].

Structural studies have shown that GroEL exists in two major conformations and that transition between the two is facilitated via nucleotide binding and release [24], [43], [57]. In the absence of nucleotide, GroEL exists in the down conformation, which does not allow for co-chaperonin binding. In this conformation, R322 in the apical domain of GroEL, which corresponds to E321 of mHsp60, forms a salt bridge with E178 in the intermediate domain. Upon nucleotide binding this salt bridge is disrupted, allowing the GroEL molecule to bind co-chaperonin and acquire an up conformation. In GroEL, mutations abolishing the formation of the salt bridge in either position, E178 or R322, were found to be suppressors for low-affinity co-chaperonin and chaperonin, respectively [51], [52]. It was suggested that elimination of this salt bridge can facilitate a shift toward the up conformation, which leads to enhanced interaction with the co-chaperonin. Although identity of residues E178 and R322 is not well conserved among chaperonins, the coupling between a positively- and a negatively- charged amino acids, which allows for formation of the salt bridge between the homologous positions, is highly conserved (Fig. S3). Indeed, restoring the salt bridge in mHsp60 by creating a double mutant E321K/K176E resulted in a wild-type phenotype (Fig. 7). Similarly, in mHsp60, disruption of this salt bridge in the E321K mutant enables mHsp60 to interact with low-affinity co-chaperonins, presumably by favoring the up conformation. This mutant forms a very stable inactive complex with mHsp10, for which dissociation of the complex is much slower and functionally irrelevant. The ability of the E321K mutant to bind mHsp10 even in the absence of nucleotides supports the suggestion that the mutant can achieve the up conformation even in the absence of ATP binding.

### The R264K/E358K Double Mutant

The second mutant that was functional with GroES harbored a double mutation. Similar to E321K, the double mutant R264K/E358K was active with GroES, both *in vivo* and *in vitro*. Nevertheless, this mutant was different since it was also active with mHsp10. Will all mutations that enable mHsp60 to function with GroES work via a mechanism of improved affinity? In contrast to the E321K mutant, the double mutant was not active with the low affinity L33A mHsp10 mutant, indicating a mechanism that does not involve global improvement of affinity for all co-chaperonins (Fig. 5). Moreover, direct binding measurements show that the increase in association equilibrium constant between mHsp10 and R264K/E358K (relative to wild-type mHsp60) was only about 1.5-fold. *In vivo* and *in vitro* examination of the two corresponding single mutants, R264K and E358K, showed that each functioned normally with mHsp10, but exhibited little, if any, activity with GroES. (Fig. 2B–E). These results suggest that positions 264 and 358 function synergistically to allow for the gain of GroES-dependent chaperone function.

What is the structural basis of the R264K/E358K phenotype? In the bacterial homologue, mutations in D359, which correspond to E358 in mHsp60, were proposed to interfere with the inter-subunit interaction of Y360 with the hydrophobic cluster A383-L183-F281 in the adjacent subunit, thereby destabilizing the down conformation [51] (Fig. S4). In a similar way, the E358K mutation in mHsp60 may interfere with the putative inter-subunit interaction between the conserved neighboring tyrosine and a similar hydrophobic cluster that exists in the adjacent mHsp60 subunit. As a result of this disruption, the up conformation would be indirectly favored. Thus, in this case, a modest increase in affinity allowed the double mutant to interact with both mHsp10 and GroES.

Furthermore, several studies pointed to D359 as a key player in allosteric transitions in GroEL [58]–[60]. In simulations of allosteric transition dynamics in GroEL subunits it was shown that the transition of GroEL to its GroES-bound conformation, is accompanied by the formation of a salt bridge between D359 of the apical domain and K80 of the equatorial domain [58], [60] (Fig. 1C). A sequence alignment shows that the homologous position to D359 of mHsp60, E358, is similarly conserved as a negatively charged residue (Fig. S5). However, the homologous mHsp60 amino acid, which corresponds to K80 of GroEL, is not fully conserved- it is K78 in some organisms, but in others it is an uncharged residue, mainly asparagine, as in humans (Fig. S5). We suggest that a salt bridge, corresponding to D359-K80, does not form in wild-type mHsp60. However, the mutation E358K in mHsp60 might enable the formation of a similar, new salt bridge between position 358 and positions E81 or E82 (Fig. S5). It is tempting to speculate that such a change may alter the allosteric signal transmitted following ATP binding and stabilize a conformation that enables interaction with GroES. Such a scenario could explain the ability of the single mutant E358 to functionally interact with GroES, albeit inefficiently (Fig. 2E). In order to examine this suggestion, we mutated positions 81 and 82 in mHsp60 to lysine, potentially enabling them to form a salt bridge with E358, and examined whether this E81K/E82K/R264K mutant displays the R264K/E358K phenotype. The triple mutant exhibited modest activity with GroES, lending support to this hypothesis. Nevertheless, the results were inconclusive because of the high background activity levels, which most likely resulted from greater oligomeric instability of this mutant (not shown).

The double mutant contains an additional mutation, which seems to function synergistically with E358. What is the significance of position 264 in mHsp60 for co-chaperonin binding? GroEL’s T266, which corresponds to R264 in mHsp60, is located in Helix I which was shown to participate in the interaction between chaperonin and co-chaperonin [43]. Moreover, amino acids adjacent to this site were shown experimentally to affect co-chaperonin binding in GroEL. For example, V264 of GroEL was shown to directly contact the GroES mobile loop in the GroEL-GroES complex crystal structure [43], [57]. The N265A mutation in GroEL was found to block GroES binding, although substrate binding in this mutant remained unimpaired (“trap” GroEL) [61]. Lastly, in a mutation correlation analysis, M267 of GroEL was shown to be related to L27 from the IVL tripeptide of the GroES mobile loop [62]. It is therefore reasonable to assume that the R264K mutation in mHsp60 locally modifies the Helix I environment, thereby improving the interaction with the co-chaperonin mobile loop. This local effect is not manifested without prior conformational changes that are induced by the E358K mutation, thereby explaining the synergistic effect of E358K and R264K.

### ADP Inhibition and Implications for the Functional Cycle of mHsp60

During the reaction cycle of the bacterial chaperonin system, following hydrolysis of ATP in the cis ring of the GroEL-GroES complex, ATP binding at the trans ring facilitates the release of GroES, ADP and substrate protein from the cis-ring [63], [64]. Since ADP can bind concomitantly to both rings of GroEL, when it is present alone or in excess over ATP, the complex between GroEL and GroES is formed, but GroES is not released from GroEL and the reaction cycle is arrested. ADP bound to the trans ring is incapable of transmitting the allosteric signal to the cis ring or of inducing the release of GroES and thus acts as an inhibitor of the folding reaction cycle [31], [55] (Fig. 8A).

A different model was proposed for the reaction cycle of the mitochondrial chaperonin system. Based upon the observation that no binding occurs between mHsp60 and mHsp10 in the presence of ADP, it was suggested that following ATP hydrolysis, when ADP occupies the cis ring, the complex between mHsp60 and mHsp10 dissociates spontaneously, without the requirement for an allosteric signal induced by ATP-binding to the trans ring [35]. How is ADP discharged from mHsp60 to allow cycling of the chaperonin? One possibility would be that ADP does not bind mHsp60 at all, and is instantly released following ATP hydrolysis and dissociation of mHsp10. However, ∼50% inhibition of the ATPase activity of the wild-type protein was observed when ADP was present in a 5-fold excess over ATP [36] indicating that ADP indeed binds to mHsp60. Another explanation can be extrapolated from our observation that ADP does not inhibit protein folding activity by wild-type mHsp60 even when it is present in a 10- fold excess over ATP (Fig. 8B). We suggest that ADP cannot occupy both rings of mHsp60 concomitantly. As a result, one of the two rings is continuously available for ATP binding, and therefore, also for mHsp10 binding. ATP binding to the unoccupied trans ring forces the release of newly-formed ADP from the cis ring, thereby recycling the chaperonin complex.

In contrast to wild-type mHsp60, we observed ADP-mediated inhibition of the protein folding activity of the two isolated mHsp60 mutants suggesting that some change has taken place either in their nucleotide binding properties or in the allosteric signal transmitted by the bound nucleotide that enables simultaneous binding of ADP to both rings. As these mutants exhibit altered co-chaperonin binding properties and possible stabilization of the up conformation, it is possible that the release of co-chaperonin from chaperonin after ATP hydrolysis is no longer spontaneous. Rather, it has now become dependent upon the allosteric signal transmitted by the binding of ATP to the trans ring and, therefore, competitive binding of ADP for the ATP sites becomes inhibitory as in the bacterial system. This suggestion is supported, at least in the case of the double mutant, by a previous prediction that the transition from up to down conformation will be most sensitive to mutations in residue D359 in GroEL [60], which corresponds to E358 in mHsp60.

Another observation which is worthy of discussion is the fact that ADP had a strong inhibitory effect on the folding activity of the double mutant only when refolding was carried out with GroES, whereas folding by the double mutant-mHsp10 pair was only mildly inhibited by ADP (Fig. 8D & G). A possible explanation for this could be that, following their binding, mHsp10 and GroES exert distinct conformational changes on the chaperonin and stabilize the ADP-bound state in the trans ring to different degrees.

### Conclusions

*In vitro* analysis of mHsp60 mutants that are functional with GroES lent insight into the molecular basis for the exclusive interaction between mHsp60 and mHsp10. Due to the dynamic character of the chaperonin machinery, much more than simple one-on-one amino acid interactions is involved in binding between chaperonin and co-chaperonin. We found that binding of co-chaperonin to chaperonin is influenced by two distinct factors. The first is the accessibility of the up conformation, which affects the general affinity of mHsp60 for co-chaperonins and the second is the nature of the allosteric signals that are transmitted upon nucleotide binding. A delicate balance between these two forces has evolved in the mitochondrial system in order to ensure an optimal interaction between chaperonin and co-chaperonin as well as to allow for additional extra-mitochondrial functions of these molecules.

## Materials and Methods

### Cloning of GroEL and GroES into a pOFX Plasmid

Using the IPTG-inducible pOFX plasmid expressing wild-type human mHsp10 and mHsp60 [8], we engineered three additional constructs containing various combinations of co-chaperonin and chaperonin from the human and bacterial chaperonin systems (Fig. S1). In order to clone GroEL into a pOFX plasmid, its ORF was amplified by standard PCR with primers 5 & 6 (Table S1) digested by restriction enzymes AflII and SpeI and ligated into pOFX which was digested with the same enzymes. The cloning of GroES into pOFX was carried out in two steps, due to cloning constraints. First, a standard PCR was carried out on the GroES ORF with primers 3 & 4 (Table S1). The PCR products were then digested with Eco105I and Eco81I and ligated into pOFX. This step resulted in GroES with an extension of 3 amino acids at its C-terminus. This extension was removed using site-directed PCR with primers 19 & 20 (Table S1).

### *In vivo* Complementation Experiment

IPTG-inducible pOFX plasmid variants were electroporated into *E. coli* strain MGM100 [67], [68]. The cells were grown on 2YT-agar plates containing 25 µg/ml Kanamycin and 50 µg/ml Spectinomycin in the presence of either: 0.2% arabinose, 0.5% glucose or 0.5% glucose and 1 mM IPTG.

### Mutagenesis and *in vivo* Screening for mHsp60 Mutants Able to Function with GroES

Random mutagenesis of mHsp60 was carried out using error-prone PCR. cDNA of mHsp60 was amplified by PCR in two steps with primers 1 & 2 (Table S1). In the first step, 4 cycles of PCR were carried out in 4 different test tubes. In each tube, the concentration of one of the dNTPs was lower (1.25 µM), compared to the concentration of the other dNTPs (0.25 mM each). In the second step, the 4 reactions were pooled together and additional 25 cycles of PCR were performed. The purified PCR products were ligated into the pOFX plasmid, already containing the GroES sequence, between the AFlII and SacI restriction sites and transformed into *E. coli* XL1-blue. Following purification from XL1-blue, the pOFX plasmids were transformed by electroporation into MGM100 competent cells. Finally, the ability of the mutant-containing bacteria to grow on 2YT-agar plates (25 µg/ml Kanamycin, 50 µg/ml Spectinomycin) was examined in the presence of 0.5% glucose and 1 mM IPTG. Colonies that were able to grow under these conditions were collected and the mHsp60 ORF was sequenced.

### Site-directed Mutagenesis

Site-directed mutants were created according to the protocol of Stratagene, using primers 7–18 (Table S1).

### Protein Purification

GroEL and GroES, wild-type and mutant proteins, were purified as previously described [69]. mHsp10(His)_6_ and mHsp60, wild-type and mutant proteins, were purified as described [70]. A Coomassie-stained gel of the purified proteins that were used in this study is shown in Fig. S2.

### Pull-down Assay

His-tagged mHsp10 (50 µM), together with 50 µM of wild-type mHsp60, E321K or GroEL were incubated for 5 min in 200 µl binding buffer composed of 50 mM Tris-HCl pH 7.7, 5% glycerol, 150 mM NaCl, 5 mM MgCl_2_, 100 mM KCl, 30 mM imidazole and nucleotides as indicated. After a 30 min incubation with 40 µl Ni-NTA beads (GE Healthcare) on an end-to-end shaker at room temperature, samples were centrifuged and washed four times with 200 µl binding buffer. The pellets, containing Ni-beads and bound proteins, were then resuspended with 200 µl sample buffer and boiled for 10 min. Equivalent aliquots of 2 µl from the total sample, the unbound fraction, fourth wash and bound (pellet) fraction were analyzed by SDS-PAGE and stained with Coomassie Brilliant Blue R-250.

### Steady-state ATPase Activity

The steady-state ATP hydrolysis was measured at 340 nm by coupling the formation of ADP to the oxidation of NADH by pyruvate kinase and lactate dehydrogenase, as previously described [71]. The reactions were carried out with 5 µM of GroEL or 10 µM of mHsp60 variants in the presence of 50 mM Na-HEPES pH 7.5, 10 mM MgCl_2_, 50 mM KCl, 0.2 mM phosphoenol pyruvate, 0.3 mM NADH, 14 units of pyruvate kinase, 7 units of lactate dehydrogenase and 2 mM ATP.

### In vitro Refolding Activity

Refolding of HCl-denatured MDH was carried out as previously described [39].

### Surface Plasmon Resonance (SPR)

The experiments were performed with a ProteOn XPR36 instrument (Bio-Rad) as previously described [65]. 0.7–3.5 µg of either GroES or mHsp10 were immobilized through amine-coupling to the GLC sensor chip (Bio-Rad). For the association phase, samples containing six concentrations of each mHsp60 variant in injection buffer were simultaneously injected over the chip. For the dissociation phase, the injection buffer was injected over the chip. The injection buffer contain 20 mM Na-HEPES pH 7.5, 100 mM KCl, 10 mM MgCl_2_, 0.005% Tween and 2 mM ATP. Apparent K_A_ values were calculated using the ProteOn Manager program (Bio-Rad).

### General Methods

The protein concentration was determined by using Sigma’s Bicinchoninic_acid protein assay with BSA as a standard (Sigma-Aldrich). Protein concentrations refer to monomer concentrations.

## Supporting Information

Figure S1 **Schematic representation of the screening procedure.** In this screen, an MGM100 strain was used, in which GroEL and GroES expression is under control of the inducible arabinose promoter P_BAD_. (A) Co-expression of mHsp60 and GroES does not allow for growth of these bacteria in the absence of arabinose. Upon co-expression of GroEL and GroES or mHsp60 and mHsp10 from an IPTG-inducible plasmid, this strain is able to grow in the presence of glucose and IPTG. (B) A library of mHsp60 mutants cloned into pOFX co-expressing GroES was transformed into MGM100. Colonies that were able to grow in the presence of glucose and IPTG were isolated and the mHsp60 open reading frame sequenced.
(DOC)

Figure S2 **SDS-PAGE of the various purified proteins used in this study.** 10 µg of each protein was separated by 14% SDS-PAGE and stained with Coomassie blue.
(DOC)

Figure S3 **The highly conserved salt bridge between positions 321/322 and 176/178.** A multiple sequence alignment of various mitochondrial and bacterial chaperonin sequences was produced using the ClustalW2 program. Only the amino acids corresponding to positions 322 and 178 in GroEL or 176 and 321 in mHsp60 are presented. Residues displaying a different charge than their counterparts in a particular position are presented with a gray background.
(DOC)

Figure S4 **Tyrosine 360 in GroEL interacts with a hydrophobic cluster of the adjacent subunit.** A three dimensional model showing a side view of a GroEL ring in the closed state. Each subunit is colored differently (left image). Y360, a neighbor of D359, interacts with the A383-L183-F281 cluster in which A383 and L183 are located on the adjacent subunit (PDB entry 1AON). Image was created using the PyMOL program.
(DOC)

Figure S5 **Multiple sequence alignment of various mitochondrial and bacterial chaperonin sequences.** Only the amino acids corresponding to positions 80 and 359 in GroEL or 70, 78, 81, 82 and 358 in mHsp60 are highlighted. Residues having a different charge from their counterparts in a particular position are presented with red letters. Produced by the ClustalW2 program.
(DOC)

Table S1 **List of primers used in this study.**
(DOC)
