# Supplementary material for: Identification of Elements That Dictate the Specificity of Mitochondrial Hsp60 for Its Co-Chaperonin
Source: PLoS One. 2012 Dec 4;7(12):e50318. doi: 10.1371/journal.pone.0050318 (PMC3514286; doi:10.1371/journal.pone.0050318)
Supplement: Figure S1 — Schematic representation of the screening procedure. In this screen, an MGM100 strain was used, in which GroEL and GroES expression is under control of the inducible arabinose promoter PBAD. (A) Co-expression of mHsp60 and GroES does not allow for growth of these bacteria in the absence of arabinose. Upon co-expression of GroEL and GroES or mHsp60 and mHsp10 from an IPTG-inducible plasmid, this strain is able to grow in the presence of glucose and IPTG. (B) A library of mHsp60 mutants cloned into pOFX co-expressing GroES was transformed into MGM100. Colonies that were able to grow in the presence of glucose and IPTG were isolated and the mHsp60 open reading frame sequenced. (DOC) [file pone.0050318.s001.doc]

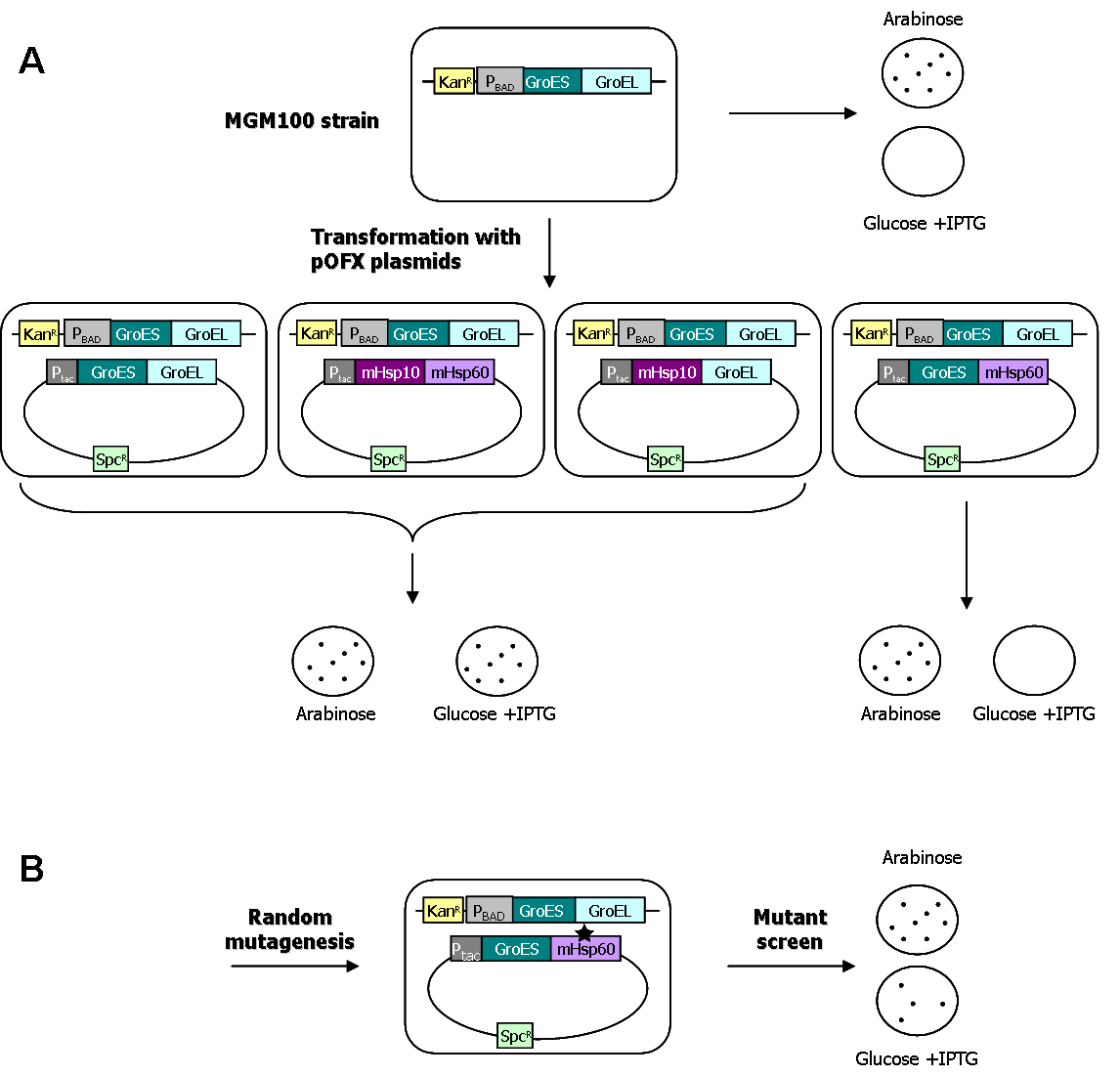


**Figure S1. Schematic representation of the screening procedure.**

In this screen, an MGM100 strain was used, in which GroEL and GroES expression is under control of the inducible arabinose promoter P_BAD_. (A) Co-expression of mHsp60 and GroES does not allow for growth of these bacteria in the absence of arabinose. Upon co-expression of GroEL and GroES or mHsp60 and mHsp10 from an IPTG-inducible plasmid, this strain is able to grow in the presence of glucose and IPTG. (B) A library of mHsp60 mutants cloned into pOFX co-expressing GroES was transformed into MGM100. Colonies that were able to grow in the presence of glucose and IPTG were isolated and the mHsp60 open reading frame sequenced.
