# Supplementary material for: Identification of Elements That Dictate the Specificity of Mitochondrial Hsp60 for Its Co-Chaperonin
Source: PLoS One. 2012 Dec 4;7(12):e50318. doi: 10.1371/journal.pone.0050318 (PMC3514286; doi:10.1371/journal.pone.0050318)
Supplement: Figure S2 — SDS-PAGE of the various purified proteins used in this study. 10 µg of each protein was separated by 14% SDS-PAGE and stained with Coomassie blue. (DOC) [file pone.0050318.s002.doc]

**Figure S2. SDS-PAGE of the various purified proteins used in this study.**

10 µg of each protein was separated by 14% SDS-PAGE and stained with Coomassie blue.
