# Supplementary material for: Identification of Elements That Dictate the Specificity of Mitochondrial Hsp60 for Its Co-Chaperonin
Source: PLoS One. 2012 Dec 4;7(12):e50318. doi: 10.1371/journal.pone.0050318 (PMC3514286; doi:10.1371/journal.pone.0050318)
Supplement: Figure S3 — The highly conserved salt bridge between positions 321/322 and 176/178. A multiple sequence alignment of various mitochondrial and bacterial chaperonin sequences was produced using the ClustalW2 program. Only the amino acids corresponding to positions 322 and 178 in GroEL or 176 and 321 in mHsp60 are presented. Residues displaying a different charge than their counterparts in a particular position are presented with a gray background. (DOC) [file pone.0050318.s003.doc]

**Figure S3. The highly conserved salt bridge between positions 321/322 and 176/178.**

A multiple sequence alignment of various mitochondrial and bacterial chaperonin sequences was produced using the ClustalW2 program. Only the amino acids corresponding to positions 322 and 178 in GroEL or 176 and 321 in mHsp60 are presented. Residues displaying a different charge than their counterparts in a particular position are presented with a gray background.
