# Supplementary material for: Identification of Elements That Dictate the Specificity of Mitochondrial Hsp60 for Its Co-Chaperonin
Source: PLoS One. 2012 Dec 4;7(12):e50318. doi: 10.1371/journal.pone.0050318 (PMC3514286; doi:10.1371/journal.pone.0050318)
Supplement: Figure S4 — Tyrosine 360 in GroEL interacts with a hydrophobic cluster of the adjacent subunit. A three dimensional model showing a side view of a GroEL ring in the closed state. Each subunit is colored differently (left image). Y360, a neighbor of D359, interacts with the A383-L183-F281 cluster in which A383 and L183 are located on the adjacent subunit (PDB entry 1AON). Image was created using the PyMOL program. (DOC) [file pone.0050318.s004.doc]

**Figure S4. Tyrosine 360 in GroEL interacts with a hydrophobic cluster of the adjacent subunit.**

A three dimensional model showing a side view of a GroEL ring in the closed state. Each subunit is colored differently (left image). Y360, a neighbor of D359, interacts with the A383-L183-F281 cluster in which A383 and L183 are located on the adjacent subunit (PDB entry 1AON). Image was created using the PyMOL program.
