# Supplementary material for: Identification of Elements That Dictate the Specificity of Mitochondrial Hsp60 for Its Co-Chaperonin
Source: PLoS One. 2012 Dec 4;7(12):e50318. doi: 10.1371/journal.pone.0050318 (PMC3514286; doi:10.1371/journal.pone.0050318)
Supplement: Figure S5 — Multiple sequence alignment of various mitochondrial and bacterial chaperonin sequences. Only the amino acids corresponding to positions 80 and 359 in GroEL or 70, 78, 81, 82 and 358 in mHsp60 are highlighted. Residues having a different charge from their counterparts in a particular position are presented with red letters. Produced by the ClustalW2 program. (DOC) [file pone.0050318.s005.doc]

**Figure S5. Multiple sequence alignment of various mitochondrial and bacterial chaperonin sequences.**

Only the amino acids corresponding to positions 80 and 359 in GroEL or 70, 78, 81, 82 and 358 in mHsp60 are highlighted. Residues having a different charge from their counterparts in a particular position are presented with red letters. Produced by the ClustalW2 program.
