# Supplementary material for: Identification of Elements That Dictate the Specificity of Mitochondrial Hsp60 for Its Co-Chaperonin
Source: PLoS One. 2012 Dec 4;7(12):e50318. doi: 10.1371/journal.pone.0050318 (PMC3514286; doi:10.1371/journal.pone.0050318)
Supplement: Table S1 — List of primers used in this study. (DOC) [file pone.0050318.s006.doc]

| **#** | **Primer name** | **Sequence 5’… 3’** |
| --- | --- | --- |
| **Primers used for error-prone PCR (restriction enzyme site is underlined):** | | |
| **1** | MEL_N_AflII | ATTCCACTGCAGCTTAAGGAGGT |
| **2** | MEL_C_SacI | ATTCGAGCTCTTGGTGAGGAA |
| **Primers used in standard PCR amplification procedure (restriction enzyme site is underlined):** | | |
| **3** | Eco81I_GroES | TATCCTAAGGAGGTGGAGTAATGAATATTCGTCCAT |
| **4** | GroES_Eco105I | TAATACGTACGCTTCAACAATTGCCAG |
| **5** | AflII_GroEL | AATCTTAAGGAGGTCGGGCTATGGCAGCTAAAGAC |
| **6** | GroEL_SpeI | TATACTAGTCTAGGAGTTACATCATGCCGCCCAT |
| **Primers used for site directed mutagenesis (mutated nucleotides are underlined):** | | |
| **7** | R264K forward | GTACACTCGTCTTGAATAAGCTAAAGGTTGGTCTTCAG |
| **8** | R264K reverse | CTGAAGACCAACCTTTAGCTTATTCAAGACGAG TGTAC |
| **9** | E358K forward | CAGTTAGATGTCACAACTAGTAAATATGAAAAGGAAAAACTG |
| **10** | E358K reverse | CAGTTTTTCCTTTTCATATTTACTAGTTGTGACATCTAACTG |
| **11** | K176E forward | GGTGTCATCACAGTAGAGGATGGAAAAACAC |
| **12** | K176E reverse | GTGTTTTTCCATCCTCTACTGTGATGACACC |
| **13** | V232L forward | CTAGTATCCAGTCCATTCTACCTGCTCTTGAAATTG |
| **14** | V232L reverse | CAATTTCAAGAGCAGGTAGAATGGACTGGATACTAG |
| **15** | L262V forward | CTAAGTACACTCGTCGTGAATAGGCTAAAGG |
| **16** | L262V reverse | CCTTTAGCCTATTCACGACGAGTGTACTTAG |
| **17** | L33A forward | CAAAGGTGGCATTATGGCTCCAGAAAAGTCTCAAG |
| **18** | L33A reverse | CTTGAGACTTTTCTGGAGCCATAATGCCACCTTTG |
| **19** | 9del_SENSE (GroES) | CTGGCAATTGTTGAAGCGTGAAATAAGTCACTATTG |
| **20** | 9del_ANTISENSE (GroES) | CAATAGTGACTTATTTCACGCTTCAACAATTGCCAG |

**Table S1. List of primers used in this study.**
